# Associations of Obesity, Physical Activity, and Screening With State-Level Trends and Racial and Ethnic Disparities of Breast Cancer Incidence and Mortality in the US

**DOI:** 10.1001/jamanetworkopen.2022.16958

**Published:** 2022-06-14

**Authors:** Zhaomin Xie, Wei Xie, Yuanke Liang, Haoyu Lin, Jundong Wu, Yukun Cui, Xuefen Su, De Zeng

**Affiliations:** 1 Guangdong Provincial Key Laboratory for Breast Cancer Diagnosis and Treatment, Cancer Hospital of Shantou University Medical College, Shantou, China; 2 Department of Medical Oncology, Cancer Hospital of Shantou University Medical College, Shantou, China; 3 Shantou University Medical College, Shantou, China; 4 Department of Breast and Thyroid Surgery, The First Affiliated Hospital of Shantou University Medical College, Shantou, China; 5 Breast Center, Cancer Hospital of Shantou University Medical College, Shantou, China

## Abstract

**Importance:**

Breast cancer causes disproportionate disease burden among various racial and ethnic groups in the US. However, state-level temporal trends and racial and ethnic disparities and whether metabolic and lifestyle factors and screening access are associated with temporal changes remain largely unknown.

**Objectives:**

To investigate temporal trends and racial and ethnic variations at the state level and ecological correlations between obesity, physical activity, and mammography screening and breast cancer incidence and mortality trends among women in the US.

**Design, Setting, and Participants:**

A cross-sectional study was conducted to analyze breast cancer incidence and mortality trends among women in the US from January 1, 1999, to December 31, 2017, whereas an ecological analysis was performed to assess the associations. Data were analyzed from March 1, 2021, to September 30, 2021. Population-based cancer registry data were obtained from US Cancer Statistics incidence and mortality data. Prevalence of obesity, physical activity, and mammography screening were obtained from the Behavioral Risk Factor Surveillance System.

**Exposures:**

Prevalence of obesity, physical activity, and mammography screening.

**Main Outcomes and Measures:**

Breast cancer incidence and mortality trends from 1999 to 2017 in the 50 US states and the District of Columbia.

**Results:**

A total of 4 136 123 breast cancer cases and 782 454 deaths were included in the analysis, with a significant reduction in incidence (average annual percent change [AAPC], −0.4% [95% CI, −0.6% to −0.2%)]) and mortality (AAPC, −1.7% [95% CI, −1.8% to −1.5%]) during the study period. A significant state-level variation in breast cancer incidence and mortality between White women and those of other races and ethnicities was observed. A significant positive correlation was found between obesity and breast cancer incidence (*r* = 0.316; *P* = .02) and mortality (*r* = 0.400; *P* = .004) and an inverse correlation was found between physical activity and incidence (*r* = −0.577; *P* < .001) in women 55 years or older and mammography screening and mortality trends (*r* = −0.644; *P* < .001) in women 40 years or older.

**Conclusions and Relevance:**

The findings of this cross-sectional study suggest that racial and ethnic disparities exist at the state level with regard to breast cancer incidence and mortality among women in the US. Metabolic and lifestyle factors and screening access were associated with the observed trends and racial and ethnic disparities. Interventions targeting these factors may help reduce the incidence of breast cancer and related deaths.

## Key Points

**Question:**

What are US breast cancer incidence and mortality trends at the state level and in each racial and ethnic group, and are metabolic and lifestyle factors correlated with these trends?

**Findings:**

This cross-sectional study using US Cancer Statistics data on more than 4 million patients with breast cancer found a significant reduction in breast cancer incidence and mortality from 1999 to 2017, with a large racial and ethnic disparity at the state level. Obesity, physical inactivity, and mammography screening were associated with the observed trends and disparities.

**Meaning:**

These findings suggest that interventions targeting metabolic and lifestyle factors and screening access may help reduce the incidence of breast cancer and related deaths, particularly among women who are members of racial and ethnic minority groups.

## Teaser

This cross-sectional study investigates state-level temporal trends of breast cancer incidence and mortality by age and racial and ethnic group and state-level ecological correlations between obesity, physical activity, and mammography screening and incidence and mortality trends among the US women.

## Introduction

In the US, breast cancer is the most commonly diagnosed cancer among women and the second leading cause of cancer-related deaths.^1^ In 2021, 281 550 new breast cancer cases were estimated to be diagnosed, corresponding to 30% of all cancers among women, and approximately 43 600 women were estimated to die of breast cancer.^2^ The American Cancer Society (ACS) reported a slight increase of 0.3% per year in invasive breast cancer incidence during the period 2012 to 2016.^3^ In contrast, a declining trend was observed in mortality rate from 1989 to 2017. Notably, a large racial and ethnic disparity in breast cancer incidence and mortality has been well documented.^1,2,3^ However, few studies have investigated comprehensively the long-term trend and racial and ethnic disparity in breast cancer incidence and mortality across the US and in each state. In addition, although the distributions of different molecular subtypes of breast cancer among racial and ethnic populations were reported by the ACS,^1^ the temporal trends of the subtypes remain less clear.

Obesity and physical activity have long been hypothesized to be associated with breast cancer incidence and mortality.^4,5,6,7^ The associations or the magnitudes of associations may differ by menopausal status and/or by race and ethnicity.^6,8^ Obesity is a well-established risk factor of postmenopausal breast cancer, particularly hormonal receptor– or estrogen receptor–positive breast cancer,^6^ because adipose tissue is an important source of estrogens after menopause.^9^ Physical activity has long been found to be protective against breast cancer.^5,7^ These associations, however, are not as well understood in women who are members of racial and ethnic minority groups as in White women.^6^ Mammography screening reduced breast cancer mortality by approximately 20% to 40% in randomized clinical trials and organized screening programs in Europe and Canada.^10,11,12,13^ To our knowledge, no study has examined whether obesity, physical activity, and mammography screening are associated with the temporal trends of breast cancer incidence and mortality at the state level in the US.

The study objectives were to investigate temporal trends of incidence and mortality of breast cancer by age and racial and ethnic group and at the state level as well as state-level ecological correlations between obesity, physical activity, and mammography screening and incidence and mortality trends in the US. The state-level analyses were performed because of the data availability at this level, because health care policies (eg, Medicaid expansion) are generally enacted by state, and because of greater power in the ecological analysis.

## Methods

Because the data used in this cross-sectional study are publicly available and deidentified, the Ethics Committee of the Cancer Hospital of Shantou University Medical College, Shantou, China, waived the need for ethics approval and informed consent from study participants. This study followed the Reporting of Observational Studies in Epidemiology (STROBE) reporting guideline.

### Data Sources

We obtained incidence and mortality data for the 50 US states and the District of Columbia for the period 1999 to 2017 from the US Cancer Statistics database maintained by the Centers for Disease Control and Prevention Wide-Ranging Online Data for Epidemiologic Research (CDC WONDER).^14^ The US Cancer Statistics database consists of publicly available data derived from the National Cancer Registry Program and the Surveillance, Epidemiology and End Results program, which includes cancer incidence and mortality data from the 50 US states, the District of Columbia, and Puerto Rico. We used leading cancer site code 26000 to select breast cancer cases and excluded in situ breast cancers. Temporal trends of breast cancer incidence by molecular subtype were also analyzed, with the available data after 2010 and in 21 Surveillance, Epidemiology and End Results areas.^15^

To investigate the state-level ecological correlations between obesity, physical activity, and mammography screening and the temporal trends of breast cancer incidence and mortality, we obtained the data from the Behavioral Risk Factor Surveillance System.^16^ The Behavioral Risk Factor Surveillance System is a national system that collects data on health-related risk behaviors via telephone interviews to monitor the health conditions of US residents and their use of health services.

### Study Variables

The study variables included age, race and ethnicity, and US state. The 3 age categories included younger than 50 years, 50 to 64 years, and 65 years and older. The CDC WONDER classification was used to cross-code race and ethnicity as Hispanic (all races), non-Hispanic American Indian or Alaska Native, non-Hispanic Asian or Pacific Islander, non-Hispanic Black or African American (hereinafter referred to as Black), and non-Hispanic White. Obesity was defined as body mass index (calculated as weight in kilograms divided by height in meters squared) of at least 30, and physical activity was defined as participating in 150 minutes or more of aerobic physical activity per week. Mammography screening rate referred to the percentage of women 40 years or older who had a mammogram within the past 2 years. Data on breast cancer incidence and mortality, obesity and physical activity prevalence, and mammography screening rates were disaggregated by year, state, age group, and race and ethnicity.

### Statistical Analysis

Incidence and mortality rates were calculated per 100 000 person-years and age-standardized to the 2000 US standard population. The Joinpoint model, developed by the Division of Cancer Control and Population Sciences of the National Cancer Institute, was used to quantify the trends of breast cancer incidence and mortality from January 1, 1999, to December 31, 2017, in the US. The annual percent change (APC), average annual percent change (AAPC), and their corresponding 95% CIs are the main parameters calculated from the underlying incidence and mortality rates using the Joinpoint Trend Analysis Software, version 4.9.0.0 (National Cancer Institute). The APC is used to evaluate the internal trend of each independent interval of the piecewise function or the global trend with 0 connection points. The AAPC is used for the comprehensive evaluation of the global mean variation trend involving multiple intervals. Pearson correlation coefficients were used to test the state-level correlations between obesity, physical activity, and screening and the AAPC of breast cancer incidence and mortality. Because obesity and physical activity have a stronger association with breast cancer incidence and mortality among postmenopausal women,^6,8^ state-level correlations between obesity and physical activity and AAPC of breast cancer incidence and mortality were performed among women 55 years or older. For the ecological associations between mammography screening and breast cancer incidence and mortality trends, the analyses were conducted among women 40 years and older. Two-sided *P* < .05 was considered statistically significant. Data were analyzed from March 1, 2021, to September 30, 2021.

## Results

### Breast Cancer Incidence Rates and Trends in the US, 1999 to 2017

A total of 4 136 123 breast cancer cases were diagnosed, yielding an incidence rate of 125.6 per 100 000 person-years (95% CI, 125.5-125.7 per 100 000 person-years) among US women during the period 1999 to 2017 (Table 1).

**Table 1.** Age-Adjusted Breast Cancer Incidence Rates and Joinpoint Trends in the US by Age and Race and Ethnicity, 1999 to 2017

Demographic characteristic	Incidence rates per 100 000 PY			No. of new cases, 1999-2017	Age-adjusted incidence rate per 100 000 PY (95% CI), 1999-2017	Trend 1/trend 3			Trend 2/trend 4			1999-2017	
	1999	2004	2017			Study years	APC (95% CI)	*P* value	Study years	APC (95% CI)	*P* value	AAPC (95% CI)	*P* value^a^
Overall	134.8	121.4	125.1	4 136 123	125.6 (125.5 to 125.7)	1999-2004	−2.1 (−2.9 to −1.4)	<.001	2004-2017	0.3 (0.1 to 0.5)	.006	−0.4 (−0.6 to −0.2)	<.05
Age, y													
<50	43.3	42.6	44.1	838 379	43.2 (43.1 to 43.3)	1999-2002	−0.9 (−2.5 to 0.7)	.23	2002-2017	0.4 (0.2 to 0.5)	<.001	0.1 (−0.1 to 0.4)	>.05
50-64	309.6	270.2	267.2	1 472 150	273.7 (273.2 to 274.1)	1999-2005	−2.8 (−3.7 to −1.9)	<.001	2005-2017	0 (−0.3 to 0.3)	.94	−0.9 (−1.3 to −0.6)	<.05
≥65	451.5	396.1	420.2	1 825 594	421.9 (421.2 to 422.5)	1999-2004	−2.8 (−3.7 to −1.8)	<.001	2004-2009	1.4 (0 to 2.8)	.06	−0.4 (−0.8 to 0.1)	>.05
						2009-2017	0.1 (−0.4 to 0.5)	.79					
Race and ethnicity^b^													
American Indian or Alaska Native	87.0	86.1	96.8	19 896	94.0 (92.7 to 95.4)	1999-2017	1.2 (0.8 to 1.6)	<.001	NA	NA	NA	1.2 (0.8 to 1.6)	<.05
Asian or Pacific Islander	86.9	84.6	99.0	135 929	90.9 (90.4 to 91.4)	1999-2004	−0.7 (−2.1 to 0.8)	.32	2004-2017	1.3 (1.0 to 1.6)	<.001	0.7 (0.3 to 1.1)	<.05
Black	119.2	117.4	124.3	438 620	123.2 (122.9 to 123.6)	1999-2005	−0.1 (−0.7 to 0.5)	.65	2005-2008	2.2 (−1.0 to 5.5)	.16	0.3 (−0.3 to 0.9)	>.05
						2008-2015	0.4 (−0.1 to 0.9)	.11	2015-2017	−1.5 (−4.5 to 1.5)	.28		
Hispanic	96.9	89.1	94.5	285 298	93.0 (92.7 to 93.4)	1999-2004	−1.6 (−2.9 to −0.2)	.03	2004-2017	0.4 (0.1 to 0.6)	.01	−0.2 (−0.6 to 0.2)	>.05
White	141.0	126.2	130.8	3 231 947	131.0 (130.8 to 131.1)	1999-2004	−2.4 (−3.2 to −1.5)	<.001	2004-2017	0.3 (0.1 to 0.5)	.003	−0.4 (−0.7 to −0.2)	<.05

Abbreviations: AAPC, average annual percent change; APC, annual percent change; NA, not applicable; PY, person-years.

^a^ Because the Joinpoint analyses did not provide an exact *P* value for AAPC, only *P* < .05 or *P* > .05 was provided.

^b^ Except Hispanic, all other races and ethnicities are non-Hispanic.

We found a significant decreasing trend in breast cancer cases (AAPC, −0.4% [95% CI, −0.6% to −0.2%]), with an annual decline of 2.1% (95% CI, −2.9% to −1.4%) from 1999 to 2004 and an annual increase of 0.3% (95% CI, 0.1%-0.5%) from 2004 to 2017. The incidence rate rose substantially with advancing age. White women had the highest incidence rate (131.0 [95% CI, 130.8-131.1] per 100 000 person-years) with a significant declining trend (AAPC, −0.4% [95% CI, −0.7% to −0.2%]), whereas a significant ascending trend was found among American Indian or Alaska Native women (AAPC, 1.2% [95% CI, 0.8%-1.6%]) and Asian or Pacific Islander women (AAPC, 0.7% [95% CI, 0.3%-1.1%]) throughout the entire study period. Twelve states showed an increasing trend, of which 2 (Mississippi and Tennessee) were significant, whereas 33 states and the District of Columbia showed a decreasing trend, with 14 being significant; there was no trend change in the remaining 5 states (eTable 1 in the Supplement). The greatest increase was observed in Mississippi (AAPC, 0.6% [95% CI, 0.2%-1.0%]), whereas the largest decline was observed in Oregon (AAPC, −1.2% [95% CI, −1.7% to −0.8%]).

Black women had the highest incidence of less favorable breast cancer subtypes, including triple-negative (12.2 per 100 000 person-years), lumina B (6.8 per 100 000 person-years), and *ERBB2* (previously *HER2* or *HER2/neu*) enriched (3.4 per 100 000 person-years). Triple-negative breast cancer was the second most common subtype among Black and Hispanic women and women 65 years or older (eTable 2 in the Supplement).

### Breast Cancer Mortality Rates and Trends in the US, 1999 to 2017

 The total number of breast cancer deaths during the study period was 782 454 in the US, with an overall mortality rate of 22.8 (95% CI, 22.7-22.8) per 100 000 person-years and a significant decreasing trend (AAPC, −1.7% [95% CI, −1.8% to −1.5%]) (Table 2). Black women had the highest mortality rate (31.5 [95% CI, 31.3-31.7] per 100 000 person-years), whereas Asian or Pacific Islander women had the lowest rate (11.8 [95% CI, 11.7-12.0] per 100 000 person-years) throughout the entire period. The largest decline in mortality was found among White women (AAPC, −1.7% [95% CI, −1.8% to −1.5%]), and the least reduction was found among Asian or Pacific Islander women (AAPC, −0.6% [95% CI, −1.0% to −0.2%]). A decreasing trend was observed in all 50 states and the District of Columbia, and only Alaska (AAPC, −1.3% [95% CI, −2.8% to 0.1%]), Mississippi (AAPC, −0.7% [95% CI, −1.8% to 0.4%]), and Tennessee (AAPC, −1.1% [95% CI, −2.4% to 0.2%]) had a nonsignificant decrease (*P* > .05) (eTable 3 in the Supplement). Of note, Connecticut (APC, 3.3% [95% CI, −6.1% to 13.6%]) and Mississippi (APC, 6.0% [95% CI, −4.4% to 17.6%]) were the only 2 states that showed a large, albeit nonsignificant, increase in mortality trend during the period 2015 to 2017.

**Table 2.** Age-Adjusted Breast Cancer Mortality Rates and Joinpoint Trends by Demographic Factors in the US, 1999 to 2017

Demographic characteristic	Mortality rates per 100 000 PY			No. of new cases, 1999-2017	Age-adjusted mortality rate per 100 000 PY (95% CI), 1999-2017	Trend 1/trend 3			Trend 2/trend 4			1999-2017	
	1999	2008	2017			Study years	APC (95% CI)	*P* value	Study years	APC (95% CI)	*P* value	AAPC (95% CI)	*P* value^a^
Overall	26.6	22.6	19.9	782 454	22.8 (22.7 to 22.8)	1999-2003	−1.4 (−1.9 to −1.0)	<.001	2003-2008	−2.3 (−2.7 to −1.8)	<.001	−1.7 (−1.8 to −1.5)	<.05
						2008- 2017	−1.4 (−1.6 to −1.3)	<.001					
Age, y													
<50	5.7	4.7	4.2	96 176	4.9 (4.9 to 5.0)	1999-2001	1.1 (−4.0 to 6.5)	.65	2001-2008	−3.1 (−4.0 to −2.1)	<.001	−1.7 (−2.3 to −1.0)	<.05
						2008- 2017	−1.1 (−1.8 to −0.5)	.002					
50-64	51.4	41.6	35.5	228 061	42.1 (41.9 to 42.3)	1999-2004	−1.6 (−2.2 to −1.1)	<.001	2004-2008	−3.2 (−4.4 to −2.0)	<.001	−2.1 (−2.4 to −1.8)	<.05
						2008-2017	−1.9 (−2.1 to −1.6)	<.001					
≥65	116.6	102.0	91.2	458 217	101.9 (101.6 to 102.2)	1999-2017	−1.4 (−1.5 to −1.4)	<.001	NA	NA	NA	−1.4 (−1.5 to −1.4)	<.05
Race and ethnicity^b^													
American Indian or Alaska Native	17.1	16.2	14.8	3056	15.6 (15.0 to 16.2)	1999-2017	−0.8 (−1.6 to −0.0)	.04	NA	NA	NA	−0.8 (−1.6 to −0.0)	<.05
Asian or Pacific Islander	12.7	11.6	11.9	16 932	11.8 (11.7 to 12.0)	1999-2017	−0.6 (−1.0 to −0.2)	.003	NA	NA	NA	−0.6 (−1.0 to −0.2)	<.05
Black	35.8	31.8	27.9	110 674	31.5 (31.3 to 31.7)	1999-2017	−1.4 (−1.5 to −1.3)	<.001	NA	NA	NA	−1.4 (−1.5 to −1.3)	<.05
Hispanic	16.6	14.6	13.5	42 392	14.8 (14.7 to 15.0)	1999-2017	−1.1 (−1.3 to −0.9)	<.001	NA	NA	NA	−1.1 (−1.3 to −0.9)	<.05
White	26.5	22.5	19.9	607 794	22.8 (22.8 to 22.9)	1999- 2012	−1.9 (−2.0 to −1.8)	<.001	2012-2017	−1.2 (−1.7 to −0.6)	.001	−1.7 (−1.8 to −1.5)	<.05

Abbreviations: AAPC, average annual percent change; APC, annual percent change; NA, not applicable; PY, person-years.

^a^ Because the Joinpoint analyses did not provide an exact *P* value for AAPC, only *P* < .05 or *P* > .05 was provided.

^b^ Except Hispanic, all other races and ethnicities are non-Hispanic.

### State-Level Racial and Ethnic Differences in Breast Cancer Incidence and Mortality

**Figure 1.**
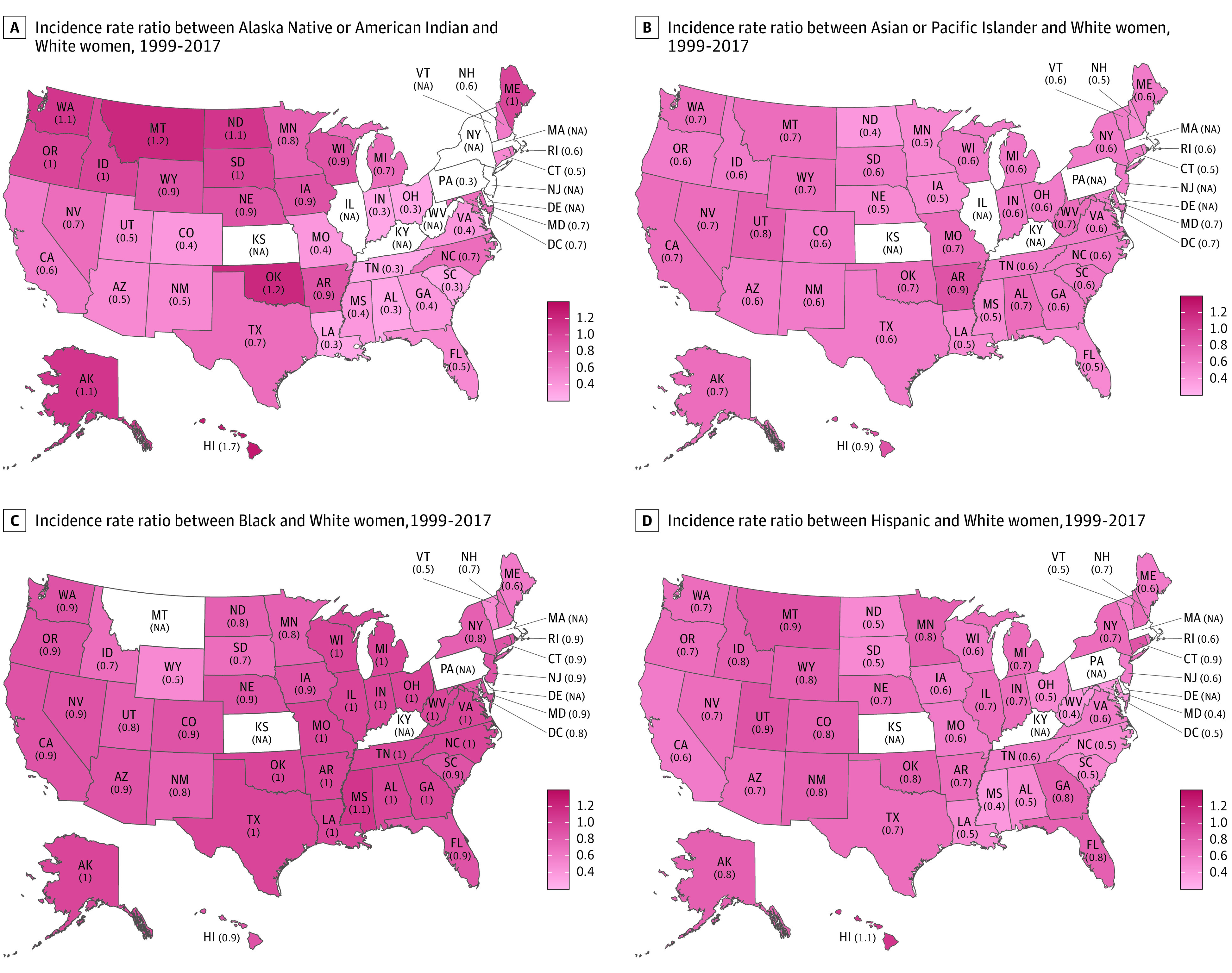
State-Level Incidence Rate Ratios Between Women in Racial and Ethnic Minority Groups and White Women, 1999 to 2017 NA indicates not applicable.

The incidence rate ratios (IRRs) for American Indian or Alaska Native vs White women varied substantially from 0.3 in the Southeast and Midwest to 1.7 in Hawaii (Figure 1A) due to the large state-to-state variation in incidence rates among American Indian or Alaska Native women (eTable 4 in the Supplement). All states had an IRR for Asian or Pacific Islander vs White women of less than 1.0 (Figure 1B). Mississippi had the highest IRR of 1.1 and Vermont, and Wyoming had the lowest IRR of 0.5 between Black and White women (Figure 1C). For the comparisons between Hispanic and White women, Hawaii had the highest IRR of 1.1, and Maryland, Mississippi, and West Virginia had the lowest IRR of 0.4 (Figure 1D).

**Figure 2.**
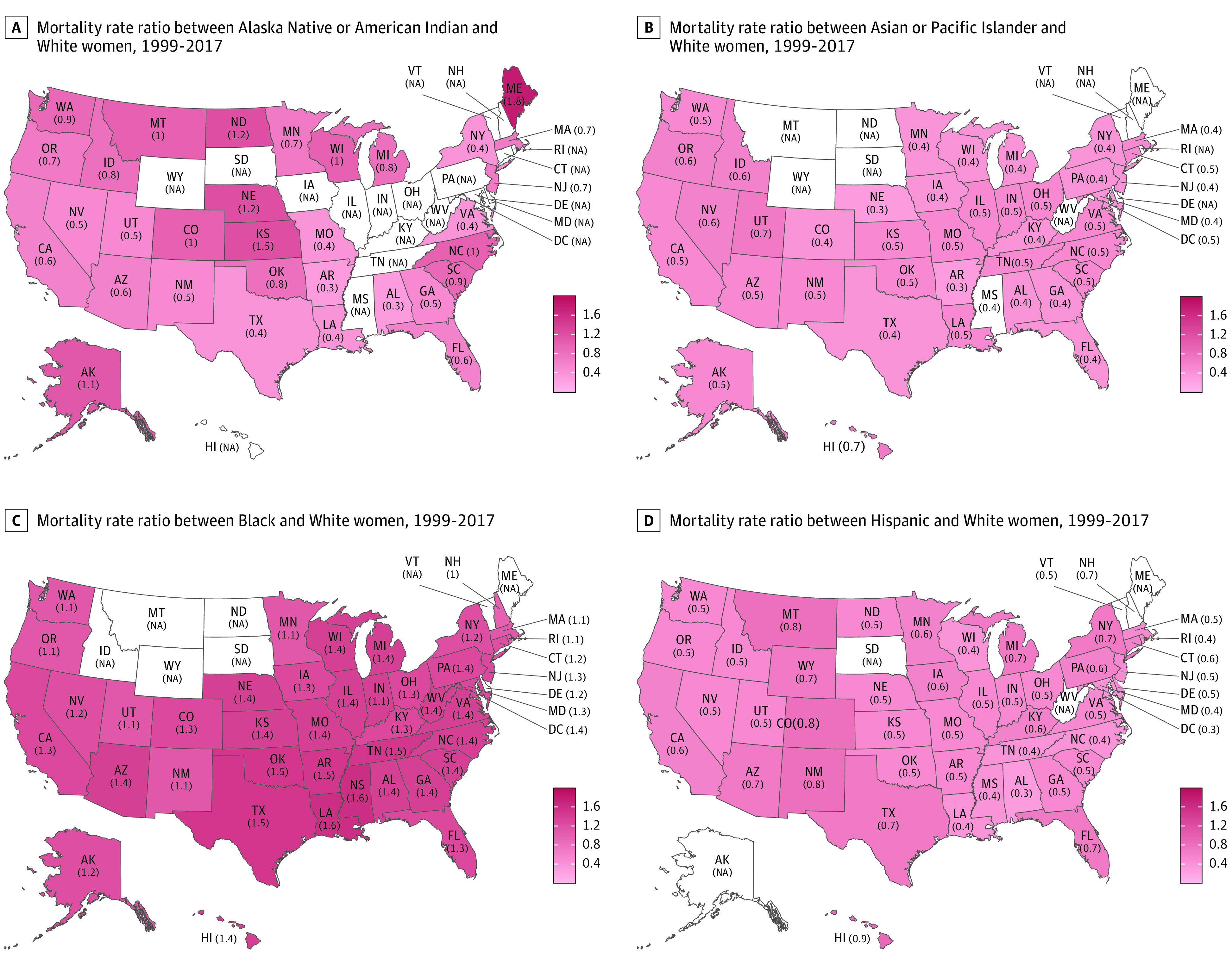
State-Level Mortality Rate Ratios Between Women in Racial and Ethnic Minority Groups and White Women, 1999 to 2017 NA indicates not applicable.

Asian and Pacific Islander and Hispanic women had a lower mortality rate than White women in every state (Figure 2B and D). Louisiana and Mississippi had the highest mortality rate ratio (MRR) of Black to White women of 1.6, whereas in New Hampshire, the rate of Black women approached that of White women (MRR of 1.0) (Figure 2C).

### Ecological Correlations Between Obesity, Physical Activity, and Mammography Screening and Breast Cancer Incidence and Mortality Trends

**Figure 3.**
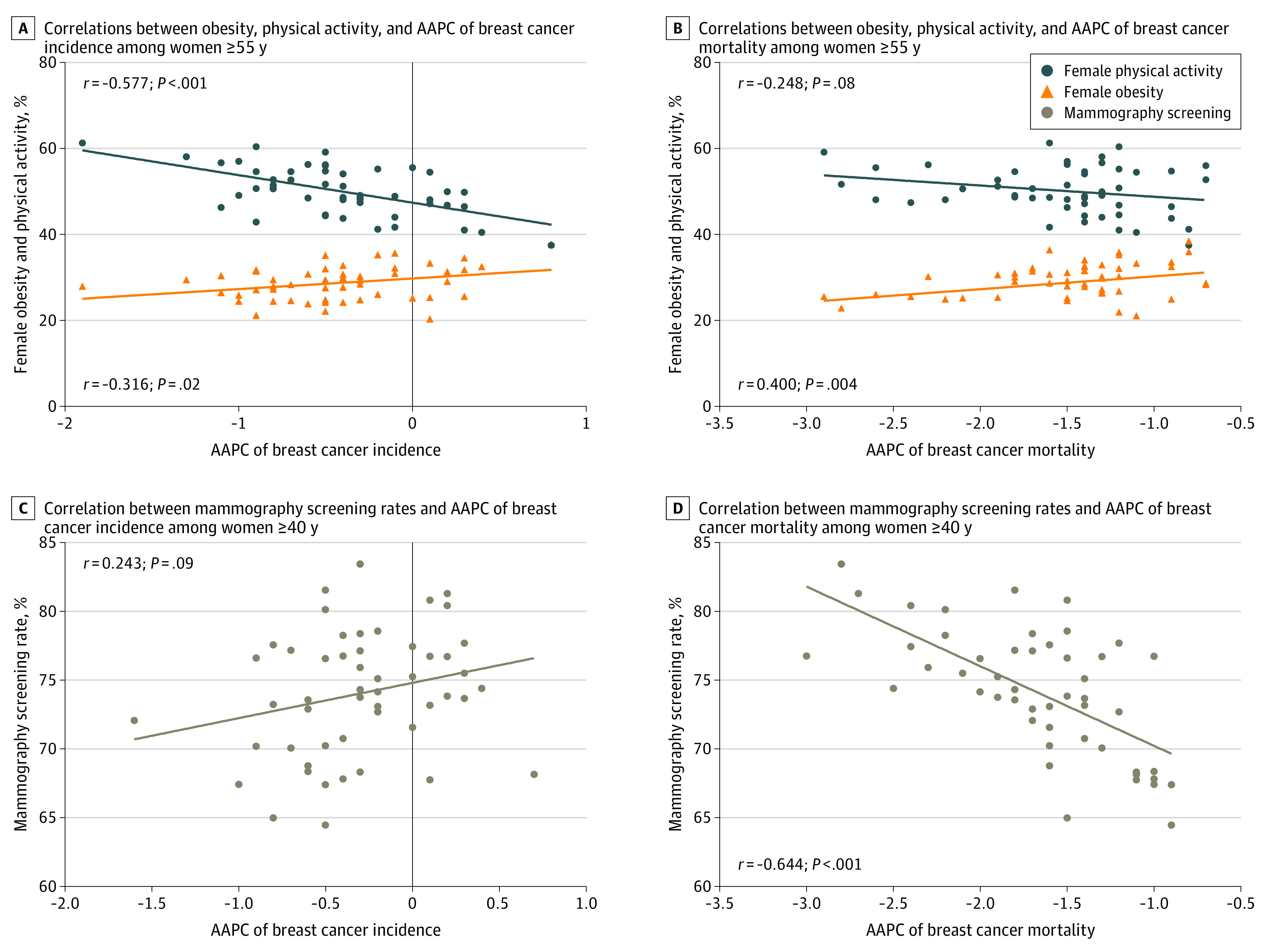
Ecological Correlations Between Obesity, Physical Activity, and Mammography Screening and Breast Cancer Incidence and Mortality Trends, 2011 to 2017 Correlations between obesity and physical activity and the average annual percent change (AAPC) of breast cancer incidence (A) and mortality (B) among women 55 years or older and between mammography screening rates and incidence (C) and mortality (D) trends among women 40 years or older. Markers indicate the 50 US states and the District of Columbia from 2011 to 2017. Obesity was defined as body mass index (calculated as weight in kilograms divided by height in meters squared) of at least 30. Physically active women were defined as women who participated in at least 150 minutes of moderate-intensity aerobic physical activity. Mammography screening was defined as having had a mammogram in the past 2 years.

In the US, the prevalence of female obesity was approximately 27.2% (95% CI, 25.3%-29.1%) in 2011 and increased to 31.5% (95% CI, 29.2%-33.7%) in 2018. The percentage of physically active women was 50.2% (95% CI, 48.0%-52.4%) in 2011 and decreased slightly to 49.7% (95% CI, 47.3%-52.1%) in 2017. Hawaii had the lowest prevalence of female obesity (20.4% [95% CI, 18.5%-22.3%]) and Oregon had the highest percentage of physically active women (61.3% [95% CI, 58.9%-63.7%]), while Mississippi had the highest prevalence of obesity (37.7% [95% CI, 35.5%-40.1%]) and the lowest percentage of physically active women (37.5% [95% CI, 35.2%-39.8%]). State-level physical activity was strongly and inversely correlated with the prevalence of obesity (*r* = −0.733; *P* < .001) (eFigure 1 in the Supplement). The mammography screening rate during the past 2 years ranged from 64.3% (95% CI, 61.8%-66.9%) in Idaho to 83.4% (95% CI, 82.0%-84.8%) in Massachusetts among women 40 years or older. We found an inverse correlation between physical activity and breast cancer incidence trend (*r* = −0.577; *P* < .001) and a positive association between obesity and breast cancer incidence trend (*r* = 0.316; *P* = .02) among women 55 years or older (Figure 3A). In the subgroup analysis by race and ethnicity, we found a positive association between obesity and AAPC of incidence among White women (*r* = 0.334; *P* = .02) and an inverse correlation between physical activity levels and incidence trend among White (*r* = −0.590; *P* < .001) and Black (*r* = −0.392; *P* = .02) women (eFigure 2 in the Supplement). Obesity was positively correlated with AAPC of breast cancer mortality in women 55 years or older (*r* = 0.400; *P* = .004) (Figure 3B), and mammography screening was inversely correlated with AAPC of breast cancer mortality at the state level in women 40 years or older (*r* = −0.644; *P* < .001) (Figure 3D).

## Discussion

In this study, a significant reduction in breast cancer incidence and mortality was found in the US from 1999 to 2017. In contrast to the continuously decreasing mortality trend, the incidence rate increased significantly from 2004 to 2017 despite a significant reduction from 1999 to 2004. The increase in incidence was mainly observed in American Indian or Alaska Native and Asian or Pacific Islander women. A striking state-level disparity between White women and those of other racial and ethnic groups was observed, with a larger difference seen in the Southeast. Finally, a positive correlation of obesity with breast cancer incidence and mortality trend and inverse associations of physical activity with incidence and mammography screening with mortality trend were observed at the state level.

An annual 2.1% reduction in breast cancer incidence from 1999 to 2004, mainly owing to the decline in Hispanic and White women,^17,18,19^ was largely attributed to the decreased use of menopausal hormones^18,19,20,21,22,23,24,25^ and a slightly lower rate of mammography screening since 2000.^26^ The increase in incidence from 2004 to 2017 may reflect a rising trend in obesity and continued declines in the fertility rate.^27^ American Indian or Alaska Native, Asian or Pacific Islander, and Black women showed a rising trend, with a significant trend among American Indian or Alaska Native women throughout the period 1999 to 2017. In our additional analyses of the breast cancer incidence trend cross-stratified by age and race and ethnicity, the results showed a significant increasing trend among women younger than 50 years in these racial and ethnic groups from 1999 to 2017 (eTable 5 in the Supplement). These findings suggest that despite the changes in screening recommendations by the US Preventive Services Task Force, American Indian or Alaska Native, Asian or Pacific Islander, and Black women younger than 50 years might still decide to obtain mammography screening. The increasing trends in breast cancer incidence might reflect the screening practice of younger women in these racial and ethnic groups. The facilitating factors might include the Affordable Care Act Medicaid expansion that extended coverage for dependents to 26 years of age and members of racial and ethnic minority groups^28^ and physicians’ recommendations.^29^ Slightly different trend changes were detected in the present study than those reported by the ACS,^1,3^ probably owing to different follow-up periods. Incidence rates in most states also showed an initially reduced trend followed by an upward trend starting from 2004.

Although the overall incidence of breast cancer in the US remains slightly higher in White women compared with Black women, the incidence rate was higher in Black women in Mississippi and approached the rate of White women in more than 30 states, findings that are slightly different from those reported by the ACS.^1^ Our results also indicate that in Hawaii, the incidence rate of Asian or Pacific Islander women is very close to that of White women, whereas the rate of Hispanic women surpassed that of White women, and the rate of American Indian or Alaska Native women was 70% higher than that in White women. Breast cancer risk factors such as age, parity, breastfeeding, reproductive and menstrual factors, obesity, physical activity, and socioeconomic status may vary by race and geographic region and partially explain the racial and geographic variations of breast cancer incidence.^30,31,32,33^

A significant reduction in breast cancer mortality overall and in most states was observed from 1999 to 2017. The magnitudes of overall change, Joinpoint trends, and declines in different populations were somewhat different from those reported by the ACS.^1,3^ Whereas we found Connecticut and Mississippi to be the only 2 states that showed a large, albeit nonsignificant, increase in mortality from 2015 to 2017, the ACS reported that Nebraska had an annual 2.5% decline from 1990 to 2010 and then a plateauing trend from 2010 to 2017.^1^ In the present analysis, the annual reduction of the death rate in Nebraska was −1.3% throughout the period 1999 to 2017.

Nationwide, the mortality disparity between Black and White women peaked in 2011—with a death rate that was 44% higher in Black women than in White women—and has been leveling off at 40% higher owing to the slower decline in the death rate among White women in recent years.^1,3^ We found that Black women had the highest incidence of the less favorable subtypes; in particular, the most aggressive subtype (triple-negative breast cancer) nearly doubled the rate found in other racial and ethnic groups. This factor may at least partially explain the highest death rate observed in Black women compared with all other racial and ethnic groups.

At the state level, breast cancer mortality rates were higher among Black women compared with White women in almost every state, with New Hampshire being the only exception. The excess death rate during the period 1999 to 2017 was 60% higher in Black women than in White women in Louisiana and Mississippi, consistent with the ACS results during 2013 to 2017.^3^ The large reduction in breast cancer mortality is mainly attributed to mammography screening and treatment advances.^34,35^ However, not all women in the US benefited from medical advances equally, because mammography screening and treatment access contributed to racial disparities in breast cancer mortality, which were largely determined by socioeconomic status. For instance, the highest disparity between Black and White women was observed in Mississippi, a state that also had one of the highest poverty rates, the highest uninsured rates, and no Medicaid expansion under the Patient Protection and Affordable Care Act.^36^ Recent studies showed that states that had Medicaid expansion reduced racial disparities in mammography screening and reduced time to treatment initiation for patients with cancer.^37^ In addition, Black women are not the only group who experience racial disparities. Reductions in mortality rates among American Indian or Alaska Native, Asian or Pacific Islander, and Hispanic women were even lower than those in Black women, and the mortality rate was 80% higher among American Indian or Alaska Native women than among White women in Maine.

To explore the underlying reasons of state-level variation in breast cancer incidence and mortality trends, we further conducted ecological analyses to examine correlations between obesity, physical activity, and mammography screening and AAPC of incidence and mortality. The results suggest that differences in the prevalence of metabolic and lifestyle risk factors and screening may at least partially explain changes in breast cancer incidence and mortality trends at the state level. There is great geographic variation in the burden of obesity and metabolic syndrome in the US, and residents living in the South are most affected.^38,39^ In addition, the states with a higher prevalence of obesity and physical inactivity were more likely to have a higher increasing trend of breast cancer incidence. For instance, Mississippi was the state with the highest prevalence of obesity and physical inactivity. Mississippi was also 1 of the 2 states that showed a significant increasing trend in breast cancer incidence and a large increase in mortality trend recently in 2015 through 2017 and had the highest MRR of Black to White women. The situation of other southern states, such as Tennessee and Louisiana, was more or less similar to that of Mississippi. These results suggest that interventions focused on promoting physical activity and reducing obesity may play important roles in addressing breast cancer disparity at the state level.

When the state-level variation was examined by race and ethnicity, the association was more apparent among White and Black women, suggesting that obesity and physical activity may have differential influences on breast cancer incidence among various racial and ethnic groups. Alternatively, data among American Indian or Alaska Native, Asian or Pacific Islander, and Hispanic women may be too sparse to detect a meaningful association. Furthermore, the states with higher mammography screening rates were more likely to have a more rapidly decreasing trend of breast cancer mortality, which confirmed the benefit of mammography screening. However, women in racial and ethnic minority groups had lower screening rates than White women,^40^ suggesting they had less access to screening services and benefited less from medical advances. It is therefore of great importance to remove the various barriers, including cultural barriers, to promote mammography screening among women in these groups.

### Limitations

This study has several limitations. Although the US Cancer Statistics database includes many incident and mortality cases of breast cancer, it lacks detailed, individual clinical and risk factor information, limiting our ability to take these factors into account when investigating mortality trends and racial and ethnic disparities and the underlying reasons. Instead, we used an ecological approach to investigate the prevalence of obesity, physical inactivity, and mammography screening and the AAPC of incidence and mortality at the state level. Despite the methodological limitations, an ecological approach is the most appropriate design to address our research hypotheses. Owing to the sparse data on American Indian or Alaska Native, Asian or Pacific Islander, and Hispanic women in some states, results for these populations may not be reliable and have limited power to detect meaningful associations.

## Conclusions

More than 20 years have passed since the National Cancer Institute funded the first series of programs to address cancer disparities in the US. The findings of this cross-sectional study suggest that large state-level variation and racial and ethnic disparities in breast cancer incidence and mortality still exist, further suggesting that the current approaches to preventing or eliminating disparities are insufficient. Novel and intensive intervention strategies on reducing obesity and promoting physical activity and mammography screening may help reduce the incidence of breast cancer and related deaths, particularly among women in racial and ethnic minority groups.

## Notes

Supplement. **eTable 1.** Age-Adjusted Breast Cancer Incidence Rates and Joinpoint Trends by US State, 1999-2017 **eTable 2.** Age-Adjusted Breast Cancer Incidence Rates and Joinpoint Trends by Molecular Subtype in the US, 2010-2018 **eTable 3.** Age-Adjusted Breast Cancer Mortality Rates and Joinpoint Trends by US State, 1999-2017 **eTable 4.** Age-Adjusted Breast Cancer Incidence Rates by State and Race and Ethnicity in the US, 1999-2017 **eTable 5.** Age-Adjusted Breast Cancer Incidence Rates by Age and Race and Ethnicity in the US, 1999-2017 **eFigure 1.** Ecological Correlation Between State-Level Obesity and Physical Activity **eFigure 2.** Ecological Correlation Between State-Level Obesity, Physical Activity, and Breast Cancer Incidence Among Women 55 Years or Older by Race and Ethnicity
