# Supplementary material for: Associations of Obesity, Physical Activity, and Screening With State-Level Trends and Racial and Ethnic Disparities of Breast Cancer Incidence and Mortality in the US
Source: JAMA Netw Open. 2022 Jun 14;5(6):e2216958. doi: 10.1001/jamanetworkopen.2022.16958 (PMC9198742; doi:10.1001/jamanetworkopen.2022.16958)
Supplement: Supplement. — eTable 1. Age-Adjusted Breast Cancer Incidence Rates and Joinpoint Trends by US State, 1999-2017 eTable 2. Age-Adjusted Breast Cancer Incidence Rates and Joinpoint Trends by Molecular Subtype in the US, 2010-2018 eTable 3. Age-Adjusted Breast Cancer Mortality Rates and Joinpoint Trends by US State, 1999-2017 eTable 4. Age-Adjusted Breast Cancer Incidence Rates by State and Race and Ethnicity in the US, 1999-2017 eTable 5. Age-Adjusted Breast Cancer Incidence Rates by Age and Race and Ethnicity in the US, 1999-2017 eFigure 1. Ecological Correlation Between State-Level Obesity and Physical Activity eFigure 2. Ecological Correlation Between State-Level Obesity, Physical Activity, and Breast Cancer Incidence Among Women 55 Years or Older by Race and Ethnicity [file jamanetwopen-e2216958-s001.pdf]

Supplementary Online Content

Xie Z, Xie W, Liang Y, et al. Associations of obesity, physical activity, and screening with state-level trends and racial and ethnic disparities of breast cancer incidence and mortality in the US. *JAMA Netw Open*. 2022;5(6):e2216958. doi:10.1001/jamanetworkopen.2022.16958

eTable 1. Age-Adjusted Breast Cancer Incidence Rates and Joinpoint Trends by US State, 1999-2017
eTable 2. Age-Adjusted Breast Cancer Incidence Rates and Joinpoint Trends by Molecular Subtype in the US, 2010-2018
eTable 3. Age-Adjusted Breast Cancer Mortality Rates and Joinpoint Trends by US State, 1999-2017
eTable 4. Age-Adjusted Breast Cancer Incidence Rates by State and Race and Ethnicity in the US, 1999-2017
eTable 5. Age-Adjusted Breast Cancer Incidence Rates by Age and Race and Ethnicity in the US, 1999-2017
eFigure 1. Ecological Correlation Between State-Level Obesity and Physical Activity
eFigure 2. Ecological Correlation Between State-Level Obesity, Physical Activity, and Breast Cancer Incidence Among Women 55 Years or Older by Race and Ethnicity

This supplementary material has been provided by the authors to give readers additional information about their work.

© 2022 Xie Z et al. *JAMA Network Open*.

eTable 1. Age-Adjusted Breast Cancer Incidence Rates and Joinpoint Trends by US State, 1999-2017

State	Incidence rates per 100 to 000 PY			No. of new cases	Incidence rates per 100 to 000 PY (95% CI)	Trend 1/Trend 3			Trend 2/Trend 4			1999-2017	
	1999	2004	2017	1999-2017	1999-2017	Years	APC (95%CI)	*P*	Years	APC (95%CI)	*P*	AAPC (95%CI)	*P*[a]
**Rising trends**													
Alabama	121.8	111.7	117.1	63689	119.3 (118.4 to 120.3)	1999-2017	0.2 (-0.1 to 0.4)	0.14				0.2 (-0.1 to 0.4)	> 0.05
Delaware	136.3	118.7	119.2	13211	130.7 (128.4 to 132.9)	1999-2017	0.1 (-0.5 to 0.6)	0.83				0.1 (-0.5 to 0.6)	> 0.05
Georgia	122.3	123.2	127	116954	124.7 (123.9 to 125.4)	1999-2017	0.1 (-0.1 to 0.4)	0.18				0.1 (-0.1 to 0.4)	> 0.05
Hawaii	136.4	122.4	144.3	19203	130.0 (128.1 to 131.9)	1999-2006	-1.6 (-3.4 to 0.2)	0.07	2006-2017	1.8 (0.9 to 2.6)	<0.001	0.4 (-0.4 to 1.2)	> 0.05
Iowa	136.2	122.3	137.2	43724	126.4 (125.2 to 127.6)	1999-2013	-0.6 (-1.1 to -0.2)	0.01	2013-2017	3.5 (0.3 to 6.7)	0.03	0.3 (-0.5 to 1)	> 0.05
Mississippi	NA	113.7	118.9	29606	115.9 (114.6 to 117.3)	2003-2017	0.6 (0.2 to 1)	0.01				0.6 (0.2 to 1)[b]	< 0.05
Nevada	106.2	110.9	118	29811	115.4 (114.1 to 116.7)	1999-2001	10.5 (0.9 to 21)	0.03	2001-2004	-6.6 (-14 to 1.5)	0.10	0.2 (-1.4 to 1.7)	> 0.05
						2004-2017	0.2 (-0.1 to 0.6)	0.20					
New Hampshire	137.8	131.8	136.3	20759	138.5 (136.6 to 140.4)	1999-2017	0.2 (-0.2 to 0.7)	0.25				0.2 (-0.2 to 0.7)	> 0.05
North Carolina	128.4	122.2	133.3	131525	129.4 (128.6 to 130.1)	1999-2003	-1.8 (-3.6 to 0)	0.05	2003-2017	0.8 (0.5 to 1)	<0.001	0.2 (-0.2 to 0.6)	> 0.05
Rhode Island	136.8	133.5	137.3	16569	133.6 (131.5 to 135.7)	1999-2017	0.2 (-0.2 to 0.7)	0.33				0.2 (-0.2 to 0.7)	> 0.05
Tennessee	118.2	111.9	118.4	83988	120.5 (119.7 to 121.4)	1999-2017	0.3 (0.1 to 0.5)	0.02				0.3 (0.1 to 0.5)	< 0.05
Virginia	129.9	125.6	121.1	106179	126.1 (125.3 to 126.8)	1999-2017	0.1 (-0.2 to 0.3)	0.59				0.1 (-0.2 to 0.3)	> 0.05
**No significant change**													
Louisiana	129	120.7	128.8	59138	122.7 (121.7 to 123.8)	1999-2005	-1.7 (-2.5 to -0.9)	0.001	2005-2017	0.9 (0.6 to 1.1)	<0.001	0 (-0.3 to 0.3)	> 0.05

© 2022 Xie Z et al. *JAMA Network Open.*

Missouri	131.3	121.9	131.9	84457	126.8 (125.9 to 127.7)	1999-2005	-1.4 (-2.4 to -0.3)	0.01	2005-2017	0.7 (0.4 to 1.1)	<0.001	0 (-0.3 to 0.4)	> 0.05
New York	134.6	124.4	134.6	283200	129.5 (129.0 to 130.0)	1999-2004	-1.3 (-2.5 to -0.2)	0.03	2004-2017	0.5 (0.2 to 0.8)	0.001	0 (-0.3 to 0.3)	> 0.05
North Dakota	134.7	122.7	129.3	9214	125.4 (122.8 to 128.0)	1999-2017	0 (-0.5 to 0.5)	0.95				0 (-0.5 to 0.5)	> 0.05
South Carolina	131.9	117.3	129.8	64700	127.0 (126.0 to 128.0)	1999-2004	-2 (-2.8 to -1.2)	<0.001	2004-2007	1.9 (-1.5 to 5.4)	0.25	0 (-0.6 to 0.5)	> 0.05
						2007-2017	0.4 (0.1 to 0.6)	0.01					
**Decreasing trends**													
Alaska	133.2	124.9	111.2	7321	127.9 (124.8 to 131.0)	1999-2017	-1 (-1.4 to -0.5)	<0.001				-1 (-1.4 to -0.5)	< 0.05
Arizona	125	114.4	109	75705	115.4 (114.5 to 116.2)	1999-2005	-2.1(-3.8 to -0.3)	0.03	2005-2017	0.2 (-0.4 to 0.7)	0.55	-0.6 (-1.2 to 0.1)	> 0.05
Arkansas	NA	110	114.6	33452	114.5 (113.2 to 115.7)	2001-2005	-3.2 (-6.7 to 0.3)	0.07	2005-2017	0.8 (0.1 to 1.4)	0.03	-0.3 (-1.2 to 0.7)[b]	> 0.05
California	137.4	120.3	121.2	453942	124.3 (123.9 to 124.6)	1999-2004	-2.4 (-3.5 to -1.2)	0.001	2004-2017	-0.1 (-0.3 to 0.2)	0.56	-0.7 (-1 to -0.4)	< 0.05
Colorado	143.3	117.2	132	62076	127.1 (126.1 to 128.1)	1999-2004	-3 (-4.5 to -1.5)	0.001	2004-2017	0.4 (0 to 0.7)	0.03	-0.6 (-1 to -0.1)	< 0.05
Connecticut	150.4	135.8	136.9	57776	139.6 (138.4 to 140.8)	1999-2003	-2.8 (-4.7 to -0.9)	0.01	2003-2017	0.3 (0 to 0.6)	0.06	-0.4 (-0.9 to 0)	> 0.05
District of Columbia	142.8	130.5	145.9	8862	143.0 (140.0 to 146.0)	1999-2017	-0.2 (-1 to 0.7)	0.66				-0.2 (-1 to 0.7)	> 0.05
Florida	134.8	114.9	113.7	276235	120.2 (119.8 to 120.7)	1999-2001	-0.3 (-3.1 to 2.6)	0.84	2001-2004	-4.8 (-7.5 to -2.1)	0.004	-0.9 (-1.4 to -0.3)	< 0.05
						2004-2015	0.4 (0.1 to 0.6)	0.004	2015-2017	-2 (-4.5 to 0.6)	0.11		
Idaho	131.8	107.6	135.9	18654	123.4 (121.7 to 125.2)	1999-2004	-3.6 (-6.4 to -0.8)	0.02	2004-2017	0.9 (0.2 to 1.5)	0.01	-0.4 (-1.2 to 0.4)	> 0.05
Illinois	138.8	121.2	132	176889	129.6 (129.0 to 130.2)	1999-2003	-3.2 (-4.6 to -1.9)	<0.001	2003-2017	0.6 (0.4 to 0.8)	<0.001	-0.2 (-0.6 to 0.1)	> 0.05
Indiana	134.2	115.5	119.5	85015	122.3 (121.5 to 123.2)	1999-2004	-2.8 (-4.4 to -1.2)	0.003	2004-2017	0.4 (0 to 0.8)	0.04	-0.5 (-1 to 0)	< 0.05
Kansas	137.1	121.4	133.3	38214	127.2 (125.9 to 128.5)	1999-2013	-0.8 (-1.3 to -0.4)	0.002	2013-2017	2.6 (-0.5 to 5.8)	0.09	-0.1 (-0.8 to 0.6)	> 0.05
Kentucky	132.3	118.6	125.7	59212	124.1 (123.1 to 125.1)	1999-2004	-1.9 (-3 to -0.8)	0.003	2004-2017	0.5 (0.3 to 0.8)	<0.001	-0.2 (-0.5 to 0.2)	> 0.05
Maine	131.4	127.1	128.4	21353	129.2 (127.4 to 131.0)	1999-2017	-0.5 (-0.7 to -0.2)	0.002				-0.5 (-0.7 to -0.2)	< 0.05
Maryland	139.1	124.5	131.1	82206	131.0 (130.1 to 131.9)	1999-2005	-1.4 (-3 to 0.2)	0.08	2005-2017	0.5 (0 to 1.1)	0.06	-0.1 (-0.7 to 0.5)	> 0.05

© 2022 Xie Z et al. *JAMA Network Open.*

Massachusetts	145.3	134.3	135.4	102973	136.9 (136.0 to 137.7)	1999-2003	-2.4 (-4.4 to -0.4)	0.02	2003-2017	0.3 (0 to 0.6)	0.05	-0.3 (-0.8 to 0.2)	> 0.05
Michigan	136.1	124.6	119	139349	125.0 (124.3 to 125.7)	1999-2005	-2 (-3.2 to -0.8)	0.003	2005-2017	0 (-0.4 to 0.4)	0.98	-0.7 (-1.1 to 0.2)	< 0.05
Minnesota	138.9	122.9	138.3	73403	131.3 (130.3 to 132.3)	1999-2004	-2.7 (-4.5 to -0.9)	0.007	2004-2017	0.6 (0.1 to 1)	0.01	-0.4 (-0.9 to 0.2)	> 0.05
Montana	143.1	119.8	137.5	14320	128.2 (126.1 to 130.4)	1999-2017	-0.3 (-0.8 to 0.3)	0.33				-0.3 (-0.8 to 0.3)	> 0.05
Nebraska	132.9	126.6	129.6	24805	127.2 (125.6 to 128.8)	1999-2010	-1.1 (-1.8 to -0.3)	0.01	2010-2017	1.1 (-0.4 to 2.6)	0.14	-0.2 (-0.9 to 0.4)	> 0.05
New Jersey	141.9	132.2	138.4	133167	133.7 (133.0 to 134.4)	1999-2003	-2.7 (-4 to -1.3)	0.001	2003-2017	0.4 (0.2 to 0.7)	<0.001	-0.3 (-0.6 to 0.1)	> 0.05
New Mexico	128.8	114.9	112.7	24302	113.3 (111.9 to 114.8)	1999-2003	-3.5 (-6 to -0.8)	0.01	2003-2017	0.2 (-0.2 to 0.5)	0.40	-0.7 (-1.3 to -0.1)	< 0.05
Ohio	137.3	123.5	130.1	166722	126.0 (125.4 to 126.6)	1999-2004	-2.4 (-3.5 to -1.2)	0.001	2004-2017	0.6 (0.3 to 0.8)	<0.001	-0.3 (-0.6 to 0.1)	> 0.05
Oklahoma	134	131.2	124.1	50114	124.6 (123.5 to 125.7)	1999-2012	-1.1 (-1.4 to -0.8)	<0.001	2012-2017	1.6 (0.3 to 2.8)	0.02	-0.4 (-0.7 to 0)	> 0.05
Oregon	154.8	132.2	119	56130	132.1 (131.0 to 133.2)	1999-2003	-3.5 (-5.5 to -1.5)	0.002	2003-2017	-0.6 (-0.9 to -0.3)	0.002	-1.2 (-1.7 to -0.8)	< 0.05
Pennsylvania	137.3	125.9	131.2	198150	129.5 (128.9 to 130.1)	1999-2003	-2.5 (-3.9 to -1.1)	0.002	2003-2017	0.5 (0.3 to 0.7)	<0.001	-0.2 (-0.5 to 0.2)	> 0.05
South Dakota	NA	114.6	125	10113	126.9 (124.4 to 129.5)	2001-2017	-0.1 (-1 to 0.9)	0.87				-0.1 (-1 to 0.9)[b]	> 0.05
Texas	126.9	114.3	112.6	267515	116.4 (116.0 to 116.9)	1999-2004	-2.1 (-3.6 to -0.5)	0.01	2004-2017	-0.3 (-0.7 to 0)	0.06	-0.8 (-1.3 to -0.4)	< 0.05
Utah	119	115.4	112.4	23935	114.1 (112.6 to 115.5)	1999-2017	-0.1 (-0.4 to 0.2)	0.42				-0.1 (-0.4 to 0.2)	> 0.05
Vermont	151.2	131.9	125.1	9762	132.4 (129.7 to 135.1)	1999-2017	-0.4 (-0.8 to 0)	0.04				-0.4 (-0.8 to 0)	< 0.05
Washington	154.1	131.7	132.9	94745	136.6 (135.8 to 137.5)	1999-2005	-2.7 (-4.3 to -1.1)	0.003	2005-2017	0.2 (-0.3 to 0.8)	0.40	-0.8 (-1.4 to -0.2)	< 0.05
West Virginia	127.6	118.3	115.3	26643	117.0 (115.5 to 118.4)	1999-2017	-0.2 (-0.6 to 0.1)	0.19				-0.2 (-0.6 to 0.1)	> 0.05
Wisconsin	139.9	118.5	133.1	80568	129.5 (128.6 to 130.4)	1999-2004	-3.2 (-4.5 to -1.9)	<0.001	2004-2017	0.6 (0.3 to 0.9)	0.001	-0.4 (-0.8 to -0.1)	< 0.05
Wyoming	133.6	117.6	107.6	6568	115.7 (112.8 to 118.5)	1999-2017	-0.8 (-1.4 to -0.2)	0.01				-0.8 (-1.4 to -0.2)	< 0.05

Abbreviations: PY, person-years; CI, confidence interval; APC, annual percent change; AAPC, average annual percent change; NA, not applicable.

[a]As the join-point analyses did not provide exact *p* value for AAPC, only $p < 0.05$ or $p > 0.05$ was provided.

[b]AAPCs were not calculated during 1999 – 2017, as incidence rates were not available in 1999. The reported AAPCs were calculated during 2001-2017 in Arkansas, 2001-2017 in South Dakota and 2003-2017 in Mississippi.

© 2022 Xie Z et al. *JAMA Network Open.*

eTable 2. Age-Adjusted Breast Cancer Incidence Rates and Joinpoint Trends by Molecular Subtype in the US, 2010-2018

Molecular subtype	Overall		Race/ethnicity (Non-Hispanic)					Age (years)			Trend 1			Trend 2		
	No. of new cases	Age adjusted per 100 000 PY	American Indian / Alaska Native	Asian / Pacific Islander	Blacks	Whites	Hispanic	< 50	50-64	≥65	years	APC (95% CI)	*P*	years	APC (95% CI)	*P*
Luminal A	533 to 629	43.8	33.6	38.8	37.5	46.4	36.0	12.7	95.8	160.4	2010-2018	1.3 (0.8 to 1.8)	< 0.01			
Triple Negative	79 to 478	6.6	5.2	5.0	12.2	6.0	6.1	2.7	15.2	18.7	2010-2018	-0.4 (-1.2 to 0.3)	0.21			
Luminal B	75 to 905	6.4	5.3	6.6	6.8	6.3	5.9	2.9	15.2	15.9	2010-2016	3.9 (2.5 to 5.2)	< 0.01	2016-2018	-4.2 (-11.0 to 3.2)	0.19
HER2-enriched	32 to 205	2.7	2.5	3.4	3.4	2.4	2.7	1.1	7.1	6.5	2010-2015	2.6 (-0.2 to 5.5)	0.06	2015-2018	-3.8 (-9.6 to 2.3)	0.15

Abbreviations: PY, person-years; CI, confidence interval; APC, annual percent change.

© 2022 Xie Z et al. *JAMA Network Open*.

eTable 3. Age-Adjusted Breast Cancer Mortality Rates and Joinpoint Trends by US State, 1999-2017

State	Mortality rates per 100 to 000 PY			No. of new cases	Age adjusted per 100 000 (95% CI)	Trend 1/Trend 3			Trend 2/Trend 4			1999-2017	
	1999	2008	2017	1999-2017	1999-2017	Years	APC (95%CI)	*P*	Years	APC (95% CI)	*P*	AAPC (95%CI)	*P*[a]
Alabama	25.7	23.3	21.2	12990	23.7 (23.2 to 24.1)	1999-2017	-1.5 (-1.9 to -1.1)	<0.001				-1.5 (-1.9 to -1.1)	< 0.05
Alaska	20.4	17.8	16.7	1102	21.2 (19.9 to 22.6)	1999-2017	-1.3 (-2.8 to 0.1)	0.07				-1.3 (-2.8 to 0.1)	> 0.05
Arizona	25.4	20.6	18	14082	20.9 (20.5 to 21.2)	1999-2017	-1.7 (-2 to -1.4)	<0.001				-1.7 (-2 to -1.4)	< 0.05
Arkansas	25.3	21.4	20.6	7741	23.0 (22.4 to 23.5)	1999-2017	-1.1 (-1.6 to -0.7)	<0.001				-1.1 (-1.6 to -0.7)	< 0.05
California	25.1	21.6	19.4	81374	21.8 (21.7 to 22.0)	1999-2017	-1.6 (-1.8 to -1.4)	<0.001				-1.6 (-1.8 to -1.4)	< 0.05
Colorado	24.8	18.9	19.3	9973	20.5 (20.1 to 20.9)	1999-2008	-2.5 (-3.7 to -1.4)	<0.001	2008-2017	-0.3 (-1.4 to 0.8)	0.57	-1.4 (-2.2 to -0.7)	< 0.05
Connecticut	25.5	21	18.3	9591	21.4 (21.0 to 21.9)	1999-2001	2.5 (-5.7 to 11.3)	0.53	2001-2015	-3 (-3.5 to -2.6)	<0.001	-1.7 (-3 to -0.5)	< 0.05
						2015-2017	3.3 (-6.1 to 13.6)	0.47					
Delaware	27.7	23.2	22.1	2448	23.5 (22.6 to 24.5)	1999-2017	-1.6 (-2.3 to -1)	<0.001				-1.6 (-2.3 to -1)	< 0.05
District of Columbia	35.8	28	24.3	1916	29.7 (28.3 to 31.0)	1999-2017	-1.6 (-2.4 to -0.7)	0.001				-1.6 (-2.4 to -0.7)	< 0.05
Florida	24.4	21.2	18.5	52178	21.3 (21.1 to 21.4)	1999-2017	-1.5 (-1.6 to -1.3)	<0.001				-1.5 (-1.6 to -1.3)	< 0.05
Georgia	26.1	22.2	21.9	21830	23.4 (23.1 to 23.7)	1999-2017	-1.1 (-1.4 to -0.8)	<0.001				-1.1 (-1.4 to -0.8)	< 0.05
Hawaii	21.1	16.3	15.6	2585	16.7 (16.0 to 17.4)	1999-2017	-1.3 (-2.2 to -0.3)	0.011				-1.3(-2.2 to -0.3)	< 0.05
Idaho	27	22	21.6	3385	21.9 (21.2 to 22.7)	1999-2017	-1 (-1.9 to -0.1)	0.03				-1 (-1.9 to -0.1)	< 0.05
Illinois	29.5	23.8	20.4	34509	24.1 (23.9 to 24.4)	1999-2017	-1.9 (-2.1 to -1.7)	<0.001				-1.9 (-2.1 to -1.7)	< 0.05
Indiana	26.4	23.9	20.9	16983	23.4 (23.1 to 23.8)	1999-2017	-1.6 (-1.9 to -1.3)	<0.001				-1.6 (-1.9 to -1.3)	< 0.05
Iowa	25.8	23.5	17.9	8131	21.2 (20.8 to 21.7)	1999-2017	-1.8 (-2.2 to -1.5)	<0.001				-1.8 (-2.2 to -1.5)	< 0.05
Kansas	25.3	21.9	18.5	7217	22.3 (21.8 to 22.8)	1999-2017	-1.8 (-2.5 to -1.2)	<0.001				-1.8 (-2.5 to -1.2)	< 0.05

© 2022 Xie Z et al. *JAMA Network Open*.

Kentucky	26.7	21.8	21.3	11514	23.4 (23.0 to 23.9)	1999-2017	-1.5 (-1.9 to -1.1)	<0.001				-1.5 (-1.9 to -1.1)	< 0.05
Louisiana	29.2	26.1	23.5	12821	26.1 (25.7 to 26.6)	1999-2017	-1.8 (-2.2 to -1.5)	<0.001				-1.8 (-2.2 to -1.5)	< 0.05
Maine	27.1	21.7	18.5	3659	20.9 (20.2 to 21.6)	1999-2017	-2.1 (-2.8 to -1.4)	<0.001				-2.1 (-2.8 to -1.4)	< 0.05
Maryland	28	25	21.6	15661	24.5 (24.1 to 24.9)	1999-2017	-1.7 (-2 to -1.4)	<0.001				-1.7 (-2 to -1.4)	< 0.05
Massachusetts	27.1	21.3	18.5	17443	21.5 (21.2 to 21.8)	1999-2017	-2.7 (-3.1 to -2.3)	<0.001				-2.7 (-3.1 to -2.3)	< 0.05
Michigan	28.1	24.3	19.2	27395	23.6 (23.3 to 23.8)	1999-2017	-1.7 (-2 to -1.5)	<0.001				-1.7 (-2 to -1.5)	< 0.05
Minnesota	24.9	21.5	16.7	12332	20.7 (20.4 to 21.1)	1999-2017	-2.2 (-2.6 to -1.8)	<0.001				-2.2 (-2.6 to -1.8)	< 0.05
Mississippi	28.1	24.9	25.5	8221	25.3 (24.8 to 25.9)	1999-2015	-1.5 (-1.9 to -1.1)	<0.001	2015-2017	6 (-4.4 to 17.6)	0.25	-0.7 (-1.8 to 0.4)	>0.05
Missouri	25.7	25	21.1	16940	24.1 (23.8 to 24.5)	1999-2017	-1.6 (-1.9 to -1.3)	<0.001				-1.6 (-1.9 to -1.3)	< 0.05
Montana	23.4	16.9	17.6	2482	21.1 (20.3 to 22.0)	1999-2017	-1.5 (-2.3 to -0.6)	0.002				-1.5 (-2.3 to -0.6)	< 0.05
Nebraska	23.9	20.7	19.7	4516	21.3 (20.6 to 21.9)	1999-2017	-1.3 (-1.8 to -0.7)	<0.001				-1.3 (-1.8 to -0.7)	< 0.05
Nevada	26.7	23.4	21.3	5909	23.4 (22.8 to 24.0)	1999-2017	-1.2 (-1.5 to -0.8)	<0.001				-1.2 (-1.5 to -0.8)	< 0.05
New Hampshire	27.6	23.6	16.1	3360	21.6 (20.8 to 22.3)	1999-2017	-2.3 (-2.9 to -1.8)	<0.001				-2.3 (-2.9 to -1.8)	< 0.05
New Jersey	28.8	25.6	20	26357	25.0 (24.7 to 25.3)	1999-2017	-2.2 (-2.5 to -1.9)	<0.001				-2.2 (-2.5 to -1.9)	< 0.05
New Mexico	23.5	19.5	20.2	4557	20.9 (20.3 to 21.5)	1999-2017	-1.1 (-1.8 to -0.4)	0.003				-1.1 (-1.8 to -0.4)	< 0.05
New York	28.3	21.9	18.1	52683	22.7 (22.5 to 22.9)	1999-2017	-2.4 (-2.6 to -2.2)	<0.001				-2.4 (-2.6 to -2.2)	< 0.05
North Carolina	26.6	22.9	21.5	23913	23.0 (22.7 to 23.3)	1999-2017	-1.4 (-1.7 to -1.1)	<0.001				-1.4 (-1.7 to -1.1)	< 0.05
North Dakota	26	22	19.5	1732	21.0 (20.0 to 22.0)	1999-2017	-2.2 (-3 to -1.3)	<0.001				-2.2 (-3 to -1.3)	< 0.05
Ohio	29.3	25.1	21.9	34907	25.1 (24.8 to 25.3)	1999-2017	-1.8 (-1.9 to -1.6)	<0.001				-1.8 (-1.9 to -1.6)	< 0.05
Oklahoma	26.4	23.1	22.3	10014	24.0 (23.5 to 24.5)	1999-2017	-1.1 (-1.5 to -0.6)	<0.001				-1.1 (-1.5 to -0.6)	< 0.05
Oregon	26.6	22	18.3	9794	22.1 (21.6 to 22.5)	1999-2017	-1.7 (-2.2 to -1.2)	<0.001				-1.7 (-2.2 to -1.2)	< 0.05

© 2022 Xie Z et al. *JAMA Network Open*.

Pennsylvania	29.2	23.8	20.7	40571	24.3 (24.0 to 24.5)	1999-2017	-1.9 (-2.2 to -1.7)	<0.001				-1.9 (-2.2 to -1.7)	< 0.05
Rhode Island	26.4	21	16.4	2879	21.2 (20.4 to 22.0)	1999-2017	-2.5 (-2.9 to -2)	<0.001				-2.5 (-2.9 to -2)	< 0.05
South Carolina	27.7	24.6	20.6	12288	23.7 (23.3 to 24.2)	1999-2017	-1.5 (-2 to -1.1)	<0.001				-1.5 (-2 to -1.1)	< 0.05
South Dakota	23.1	23.3	17.5	2067	21.2 (20.3 to 22.2)	1999-2017	-1.6 (-2.5 to -0.7)	0.002				-1.6 (-2.5 to -0.7)	< 0.05
Tennessee	26.1	22.7	21	17009	23.7 (23.4 to 24.1)	1999-2005	0.1 (-1.3 to 1.5)	0.10	2005-2008	-4.7 (-12.2 to 3.5)	0.22	-1.1 (-2.4 to 0.2)	> 0.05
						2008-2017	-0.7 (-1.4 to 0.1)	0.08					
Texas	25.2	21.9	19.8	50214	21.9 (21.7 to 22.1)	1999-2017	-1.6 (-1.7 to -1.4)	<0.001				-1.6 (-1.7 to -1.4)	< 0.05
Utah	21.7	20.5	20.2	4490	21.5 (20.8 to 22.1)	1999-2017	-1 (-1.6 to -0.5)	0.001				-1 (-1.6 to -0.5)	< 0.05
Vermont	25.4	16.9	17.3	1635	21.0 (20.0 to 22.1)	1999-2017	-2.8 (-3.8 to -1.8)	<0.001				-2.8 (-3.8 to -1.8)	< 0.05
Virginia	27.2	23.8	21.8	20556	24.0 (23.7 to 24.4)	1999-2017	-1.7 (-2 to -1.5)	<0.001				-1.7 (-2 to -1.5)	< 0.05
Washington	23.9	21.1	19.3	15273	21.5 (21.2 to 21.9)	1999-2017	-1.4 (-1.8 to -1.1)	<0.001				-1.4 (-1.8 to -1.1)	< 0.05
West Virginia	24.8	23.4	22.2	5595	23.3 (22.6 to 23.9)	1999-2017	-1.1 (-1.6 to -0.5)	0.001				-1.1 (-1.6 to -0.5)	< 0.05
Wisconsin	26.6	20.6	17.3	14432	21.6 (21.3 to 22.0)	1999-2017	-1.9 (-2.2 to -1.7)	<0.001				-1.9 (-2.2 to -1.7)	< 0.05
Wyoming	23	16.8	16.6	1200	20.8 (19.6 to 22.0)	1999-2017	-1.7 (-2.8 to -0.6)	0.004				-1.7 (-2.8 to -0.6)	< 0.05

Abbreviations: PY, person-years; CI, confidence interval; APC, annual percent change; AAPC, average annual percent change.

[a]As the join-point analyses did not provide exact p value for AAPC Only p < 0.05 or p > 0.05 was provided.

© 2022 Xie Z et al. *JAMA Network Open*.

eTable 4. Age-Adjusted Breast Cancer Incidence Rates by State and Race and Ethnicity in the US, 1999-2017

State	White	Black	American Indian or Alaska Native	Asian or Pacific Islander	Hispanic
Alabama	119.2	120	37.1	81.4	62
Alaska	128.8	125.5	146.4	87.3	108
Arizona	123.1	108.1	64.1	77.1	91.7
Arkansas	114.2	112.9	104.1	97.1	82.2
California	142.4	129.2	86.6	98	90.4
Colorado	131.5	115.7	52.9	75.8	103.8
Connecticut	143.3	122.2	74.1	84.3	NA
Delaware	NA	NA	NA	NA	NA
District of Columbia	166.8	135.8	115.2	85.8	81
Florida	126	109.7	62.9	67.1	102.4
Georgia	127.4	123.7	51.2	77.3	100.9
Hawaii	136.3	117.8	237.3	127	148.4
Idaho	125.1	92.7	119.2	81.2	93.9
Illinois	134.8	128.8	NA	NA	90.4
Indiana	123.4	122.7	36.6	73.3	85.9
Iowa	127.8	114.3	113	69.4	70.4
Kansas	NA	NA	NA	NA	87.8
Kentucky	NA	NA	NA	NA	NA
Louisiana	123.4	129.1	31	60.5	59.4
Maine	129.6	79.7	131.4	79.1	75.6
Maryland	137.5	129.2	89.9	82.7	61.3
Massachusetts	NA	NA	NA	NA	111.1
Michigan	125.9	123.4	94.2	78.8	89
Minnesota	133	103.9	109.7	69.8	109.7
Mississippi	114.3	121	51.2	61	49.5
Missouri	127.3	131.3	52.5	82.8	80
Montana	127.5	NA	146.7	92.6	118.5
Nebraska	129.3	118	112.1	59.9	96.6
Nevada	123.9	112.8	88	83.3	83.8
New Hampshire	139.6	103.8	88.4	72.2	95.9
New Jersey	145.5	127.2	NA	93.5	84.9
New Mexico	127.1	99.2	69	72.8	99.9

© 2022 Xie Z et al. *JAMA Network Open*.

New York	140.7	115.6	NA	88.9	99.8
North Carolina	132.4	128.3	87.4	74.7	62.8
North Dakota	125.5	99.9	134.9	50.9	60.7
Ohio	126.8	124	34.2	75.1	67
Oklahoma	123	127.7	147.7	87.2	101.1
Oregon	134.1	121.9	127.5	84.2	100.2
Pennsylvania	NA	NA	NA	NA	94.1
Rhode Island	137.1	116.6	78.6	67.9	77.5
South Carolina	129.7	122.8	44.5	78.4	70.9
South Dakota	127.8	92.8	126.9	81.3	65.9
Tennessee	121.3	121.1	35	74.8	74.2
Texas	127.5	121.7	93.6	71.8	91.2
Utah	116.1	92.6	53.7	88.3	101.4
Vermont	133.3	60.8	NA	81.4	67.4
Virginia	129	129	47.8	75.2	81.2
Washington	140.8	121.6	148.3	92.8	100.6
West Virginia	117.5	114.9	NA	87.1	50
Wisconsin	130.8	127.5	122	73.3	83.1
Wyoming	117.6	64.6	109.4	85.6	90.9

© 2022 Xie Z et al. *JAMA Network Open*.

eTable 5. Age-Adjusted Breast Cancer Incidence Rates by Age and Race and Ethnicity in the US, 1999-2017

Demographic factors		Trend 1/Trend 3			Trend 2/Trend 4		
Age (years)	Race/ethnicity (Non-Hispanic)	years	APC (95%CI)	P Value	years	APC (95%CI)	P Value
< 50	American Indian / Alaska Native	1999-2017	2.0 (1.3 to 2.6)	< 0.001			
	Asian / Pacific Islander	1999-2017	1.2 (1.0 to 1.4)	< 0.001			
	Blacks	1999-2017	0.3 (0.1 to 0.5)	< 0.001			
	Whites	1999-2002	-1.0 (-2.2 to 0.2)	0.09	2002-2017	0.6 (0.5 to 0.7)	< 0.001
	Hispanic	1999-2010	-0.5 (-0.9 to -0.0)	0.04	2010-2017	1.1 (0.4 to 1.8)	0.006
50-64	American Indian / Alaska Native	1999-2017	0.6 (0.0 to 1.2)	< 0.001			
	Asian / Pacific Islander	1999-2006	-1.3 (-2.5 to 0.0)	0.06	2006-2017	1.6 (1.1 to 2.1)	< 0.001
	Blacks	1999-2015	0.5 (0.3 to 0.7)	< 0.001	2015-2017	-1.4 (-5.8 to 3.3)	0.53
	Whites	1999-2001	-0.1 (-4.2 to 4.1)	0.94	2001-2004	-4.8 (-8.5 to -1.0)	0.02
		2004-2012	-0.3 (-0.9 to 0.2)	0.20	2012-2017	0.6 (-0.3 to 1.5)	0.15
	Hispanic	1999-2010	-0.8 (-1.2 to -0.4)	< 0.001	2010 2017	1.0 (0.4 to 1.7)	0.004
≥65	American Indian / Alaska Native	1999-2015	1.9 (1.2 to 2.5)	< 0.001	2015 2017	-7.6 (-18.8 to 5.2)	0.21
	Asian / Pacific Islander	1999-2005	-1.3 (-2.5 to -0.0)	0.05	2005-2008	4.0 (-2.4 to 10.9)	0.2

© 2022 Xie Z et al. *JAMA Network Open*.

		2008-2017	0.9 (0.5 to 1.4)	< 0.001			
	Blacks	1999-2004	-0.4 (-1.3 to 0.6)	0.43	2004-2009	2.5 (1.2 to 3.8)	< 0.001
		2009-2017	0.4 (-0.0 to 0.8)	0.055			
	Whites	1999-2004	-2.9 (-3.9 to -1.9)	< 0.001	2004-2009	1.3 (-0.1 to 2.8)	0.07
		2009-2017	0.2 (-0.2 to 0.7)	0.30			
	Hispanic	1999-2004	-1.3 (-2.6 to 0.1)	0.08	2004-2017	0.6 (0.3 to 0.8)	< 0.001

Abbreviations: CI, confidence interval; APC, annual percent change.

© 2022 Xie Z et al. *JAMA Network Open*.

eFigure 1. Ecological Correlation Between State-Level Obesity and Physical Activity

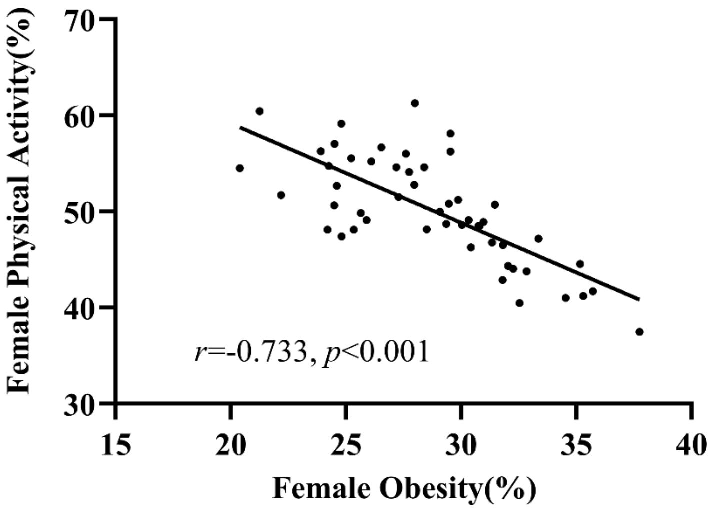

Each dot represents a US state and the DC. Obesity was defined as body mass index⩾30 kg/m^2. physically active was defined as participating in 150 minutes or more of aerobic physical activity per week.

© 2022 Xie Z et al. *JAMA Network Open*.

eFigure 2. Ecological Correlation Between State-Level Obesity, Physical Activity, and Breast Cancer Incidence Among Women 55 Years or Older by Race and Ethnicity

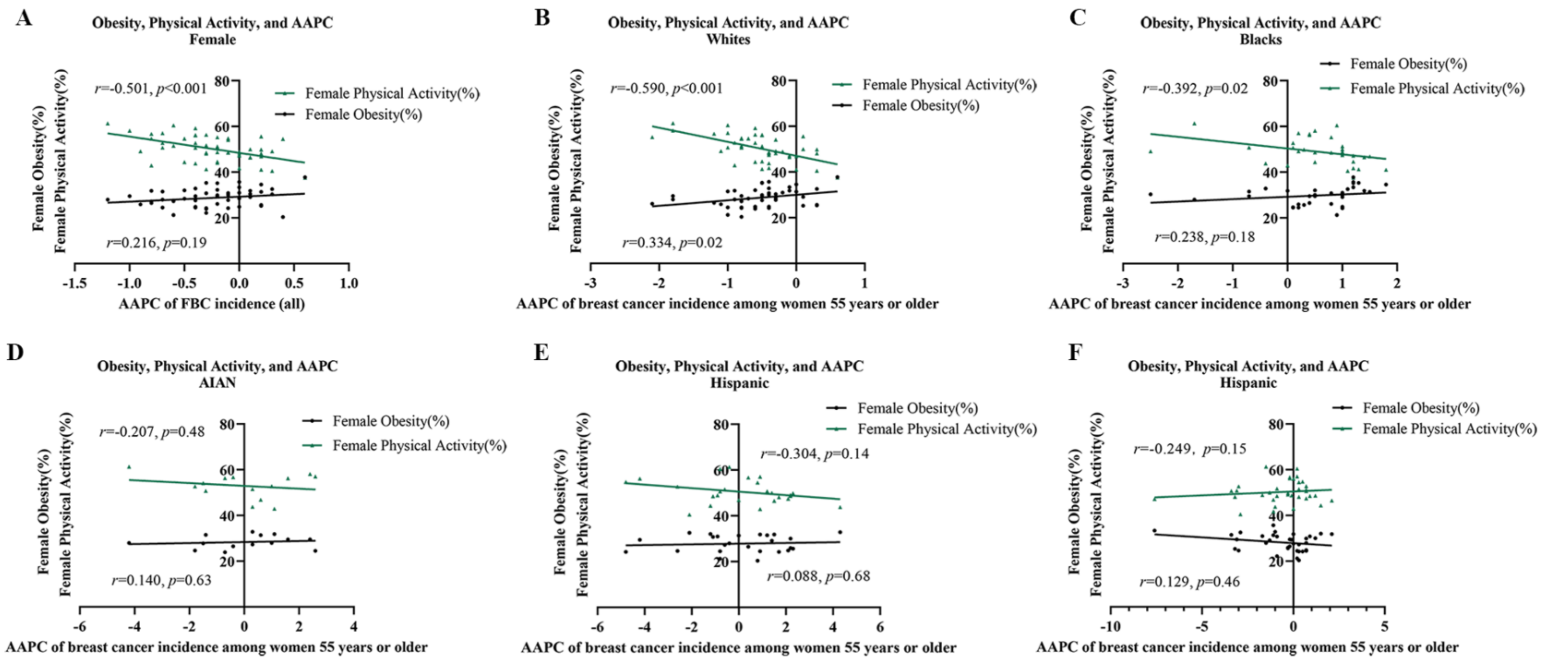

The *x* axis represents the AAPC of breast cancer incidence among women aged 55 or older and in each racial/ethnic group. Black dots represent the mean state-level percent of obesity during 2011-2017, and green triangles represent the mean state-level percent of physically active women during 2011-2017. Each dot and triangle represent a US state and the DC. AI/AN: American Indian/Alaska Native; API: Asian and Pacific Islander.

© 2022 Xie Z et al. *JAMA Network Open*.
